# DNA spontaneously wrapping around a histone core prefers negative supercoiling: A Brownian dynamics study

**DOI:** 10.1371/journal.pcbi.1012362

**Published:** 2025-01-28

**Authors:** Chunhong Long, Hongqiong Liang, Biao Wan

**Affiliations:** 1 School of Science, Chongqing University of Posts and Telecommunications, Chongqing, China; 2 Wenzhou Institute, University of Chinese Academy of Sciences, Wenzhou, Zhejiang, China; OvGU; Medical Faculty, GERMANY

## Abstract

In eukaryotes, DNA achieves a highly compact structure primarily due to its winding around the histone cores. The nature wrapping of DNA around histone core form a 1.7 left-handed superhelical turns, contributing to negative supercoiling in chromatin. During transcription, negative supercoils generated behind the polymerase during transcription may play a role in triggering nucleosome reassembly. To elucidate how supercoils influence the dynamics of wrapping of DNA around the histone cores, we developed a novel model to simulate the intricate interplay between DNA and histone. Our simulations reveal that both positively and negatively supercoiled DNAs are capable of wrapping around histone cores to adopt the nucleosome conformation. Notably, our findings confirm a strong preference for negative supercoiled DNA during nucleosome wrapping, and reveal that the both of the negative writhe and twist are beneficial to the formation of the DNA wrapping around histone. Additionally, the simulations of the multiple nucleosomes on the same DNA template indicate that the nucleosome tends to assemble in proximity to the original nucleosome. This advancement in understanding the spontaneous formation of nucleosomes may offer insights into the complex dynamics of chromatin assembly and the fundamental mechanisms governing the structure and function of chromatin.

## Author summary

The compacted organization of DNA within chromatin is primarily attributed to its intricate winding around histone cores. This winding process involves 1.7 left-handed superhelical turns around the histone core, contributing to the negative supercoiling within chromatin fibers. To gain deeper insights into how DNA supercoiling impacts the dynamics of DNA wrapping around histone cores, we first devised a novel computational model that simulates the intricate interplay between DNA and histone cores. The simulations reveal that both of the positively and negatively supercoiled DNA can spontaneously adopt the nucleosome conformation upon wrapping around the histone core, and demonstrated a strong preference for negative supercoiling during nucleosome wrapping. Additionally, the simulations of multiple nucleosomes assembling show that the nucleosome tends to assemble near original nucleosome. Finally, we examined that both the negative writhe and twist components are advantageous for the formation of the nucleosome. The studies shed light on the intricate dynamics underlying chromatin assembly and its functional implications.

## Introduction

In eukaryotes, the primary step in the intricate folding of DNA into chromatin involves the wrapping of DNA strands around the histone core[1]. The crystal structure of the histone core particle of chromatin contains two copies of each histone protein, H2A, H2B, H3 and H4 being assembled into an octamer that accommodates approximately about 145 base pairs (bp) wrapped around it [2–5]. The negatively charged DNA wraps around the positively charged histone proteins about two turns in a counter-clockwise fashion [6,7].The left-handed wrapping of DNA around the histone octamer results in the formation of approximately 1.7 superhelical turns [1,8]. The packaging of DNA within a robust nucleosome framework in eukaryotes physically obstructs the establishment of the initial replication and transcription complexes, as well as the subsequent activities of the respective machineries. Conversely, during transcription elongation, the positive supercoils generated in front of the RNA polymerase may destabilize the nucleosome while the negative supercoils behind the polymerase may aid in triggering the nucleosome reassembling [9,10]. Recent biochemical and molecular biological experiments have focused on stretching DNA molecules to investigate their mechanical or dynamical behaviors [6,11–14]. Meanwhile, some theoretical works have also explored the mechanical or dynamical properties of DNA and chromatin [15–17]. Along with experimental and theoretical studies of DNA and chromatin, numerous molecular dynamics simulation studies have been done on structures and stabilities of nucleosomes [18–25]. Recently, the Brownian dynamics methods have been developed to study the kinetics of DNA and nucleosome interactions, providing further insights into the dynamic nature of chromatin assembly and function [15,26–32].

In this study, we developed a Brownian dynamics model to investigate the dynamics properties of spontaneously nucleosome wrapping by supercoiled DNA. Our model integrates a discrete worm-like chain and a rotational sphere, for representing DNA and histone, respectively. Employing this model, we confirm that the interaction between histone and DNA is the primary driving force behind nucleosome wrapping, regardless of the supercoiling of the DNA. Importantly, our simulations have demonstrated that negative supercoils are much more beneficial to spontaneous nucleosome wrapping, which supports that negative supercoils facilitate the wrapping of DNA around histone octamers, thereby promoting the formation of nucleosomes. In addition, the simulations of the multiple nucleosomes on the same DNA template reveal that the histone core tends to assemble near the last nucleosome. Finally, we discussed the mechanisms underlying the preference for negative supercoiling during nucleosome wrapping. Our findings thereby offer insights into how DNA supercoiling affects chromatin structure, function, and assembly. Our model paves the way for studying how DNA manipulations by magnetic beads in single molecule techniques and supercoiling modifications by enzymes [13,14,33,34], such as polymerases, gyrase, and Topo II, influence and regulate chromatin properties.

## Method

**Fig 1:**
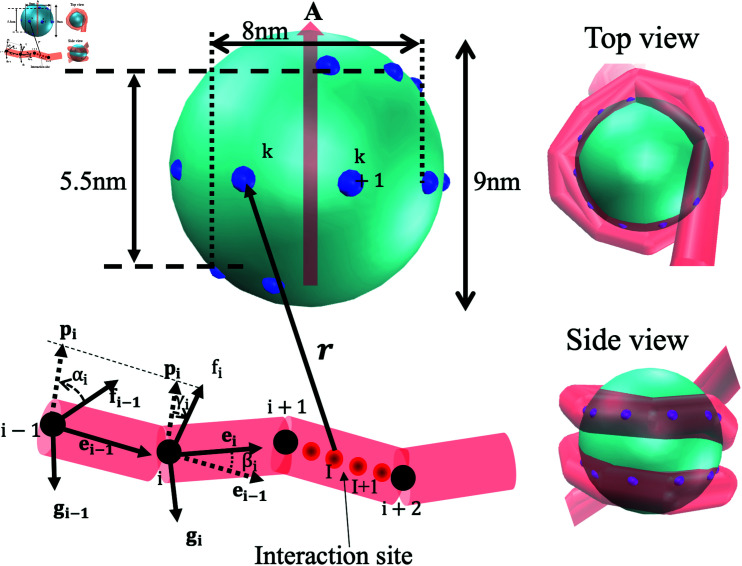
Schematic view of histone-DNA interaction model. The histone core is modeled as a sphere entity, possessing 22 interaction points along a left-handed helical path that enable the DNA to be wound around the histone core. The axial vector **A** of the helical path on the sphere describes the orientation of the histone core. The DNA is modeled as *N* discrete segements. The *i*-th segment is defined by vertices *i* and *i* + 1, attached by $f_{i}$, $g_{i}$ and $e_{i}$. The vectors define the bending angle $\beta_{i}=\arccos(e_{i-1}\cdot e_{i})$, and the twisting angle $\theta_{i}=\alpha_{i}+\gamma_{i}$ by introducing an auxiliary vector $p_{i}=e_{i-1}\times e_{i}$. (Right) Two views of a nucleosome structure generated by VMD from the simulations.

Our coarse grained model consists of a discrete worm-like chain and a rotational sphere, depicted in Fig 1, to simulate the supercoiled DNA and the histone core, respectively.

The discrete worm-like chain is comprised of *N* jointed discrete segments (cylinders) [35]. The conformation of this chain is described by *N* + 1 vertices $\left\{r_{i}\right\}$. The i-th segment is defined by the vector $s_{i}\equiv r_{i+1}\;-\;r_{i}$. The equilibrium length of each segment is $l_{0}=3.4\;\text{nm}$, representing 10 bp. In addition, an orthogonal base vector frame $\left\{e_{i},f_{i},g_{i}\right\}$ ($e_{i}=f_{i}\;\times \;g_{i}$) is attached to the segment (Fig 1), where $e_{i}\equiv s_{i}∕s_{i}$, $f_{i}$ and $g_{i}$ describe a local rotational angle $\varphi_{i}$ of the segment about its axis $e_{i}$. The torsion of the chain is described by *N* rotational angles $\left\{\varphi_{i}\right\}$. The i-th bending angle $\beta_{i}$ is defined as $\arccos(e_{i-1}\cdot e_{i})$. The i-th twist $\theta_{i}$ depends on the two frames $\left\{f_{i-1},g_{i-1},e_{i-1}\right\}$ and $\left\{f_{i},g_{i},e_{i}\right\}$. By introducing an auxiliary vector $p_{i}=e_{i-1}\;\times \;e_{i}$, the twist is $\theta_{i}=\alpha_{i}+\gamma_{i}$, where $\alpha_{i}$ is the rotation angle from $f_{i-1}$ towards $p_{i}$, $\gamma_{i}$ is the angle from $p_{i}$ towards $f_{i}$.

The stretching, bending, twisting potential energies are all harmonic [35]. The electrostatic interaction is represented as the Debye-Hückel potential on the uniformly distributed point-like charges on the segments [36–38]. Thus the energetics of the DNA chain is


$$\begin{array}{c} E=\overset{N-1}{\underset{i=0}{\sum }}\frac{\;{\text{k}}_{\text{B}}\text{T}}{2{\left(l_{0}\alpha_{s}\right)}^{2}}{\left(l_{0}-s_{i}\right)}^{2}+\overset{N-1}{\underset{i=1}{\sum }}\;{\text{k}}_{\text{B}}\text{T}\alpha_{b}\beta_{i}^{2}+\overset{N-1}{\underset{i=1}{\sum }}\frac{\alpha_{t}}{2l_{0}}\theta_{i}^{2}+\frac{\nu^{2}l_{0}^{2}}{\lambda^{2}D_{e}}\overset{\lambda N}{\underset{I=0}{\sum }}\overset{\lambda N}{\underset{J=I+N_{near}+1}{\sum }}\frac{exp(-\kappa r_{IJ})}{r_{IJ}}, \end{array}$$ (1)

where the first term on the right-hand side is the stretching energy of each segment and $\alpha_{s}$ is a stiffness coefficient; the second term is the bending energy and $\alpha_{b}$ is a bending stiffness coefficient; the third one is the twisting energy and $\alpha_{t}$ is a torsional stiffness coefficient [35]; the last one is the electrostatics and *ν* and *λ* are the effective charge density on each segment and the number of the point-like charges per segment, respectively, $D_{e}$ is the dielectric constant of water, $N_{near}$ is the number of neighbor point-like charges of each segment, and *κ* is the inverse Debye length. Moreover, the impenetrability of DNA segments is guaranteed by introducing a repulsion force $F_{IJ}^{ex}=-F_{ex}\frac{r_{IJ}}{r_{IJ}}$ if $r_{I,J}<2\;\text{nm}$ [36], where the indices *I* and *J* denote the point-like charges, not the vertices. All the simulation parameters used in this method are listed in the Table 1.

**Table 1:** Constants and parameters.

Parameter	Symbol	Value [35,37]
Number of segments	*N*	100
Temperature	*T*	298K
Histone core radius	$R_{h}$	4 . 5nm
Segment equilibrium length	$l_{0}$	3 . 4nm
Stretching stiffness coefficient	$\alpha_{s}$	0 . 1
Bending stiffness coefficient	$\alpha_{b}$	7 . 32
Torsional stiffness coefficient	$\alpha_{t}$	$400\;\text{pN}\cdot {\text{nm}}^{2}$
Monovalent salt concentration		150 mM
Dielectric constant	$D_{e}$	$8.9\times 10^{-9}\;\text{F/m}$
Effective charge density	$\rho_{e}$	8 . 01e/nm
Inverse Debye length	*κ*	$1.261\;{\text{nm}}^{-1}$
Number of the point charges per segment	*λ*	5
Electrostatic cutoff distance	$r_{c}$	8nm
DNA excluded volume force	$F_{ex}$	15pN
Viscosity	*η*	0 . 001kg/(m ⋅ s )
Hydrodynamic DNA radius	$R_{H}$	1 . 3nm
Hydrodynamic DNA rotational radius	$R_{d}$	1 . 2nm
Simulation time-step size	*Δt*	25 ps

The histone core is considered as a rotational sphere with diameter 9nm, on which 22 discrete interaction points are distributed along a left-handed helical path, spanning approximately 2 turns. The helical path of these interaction sites extends vertically for a height of 5.5 nm, while the diameters of the helix’s apical and basal termini approximate 8 nm.

The interactions between the DNA and the points on the sphere are modeled as the Morse potential [29]


$$\begin{array}{c} U_{DH}=\underset{k,I}{\sum }\epsilon \left[{\left(e^{-\alpha (r-\delta )}-1\right)}^{2}-1\right] \end{array}$$ (2)

where *ϵ* is the absorption energy, e.g, chosen as $5\;{\text{k}}_{\text{B}}\text{T}$ (corresponding to the absorption energy density 8$\;{\text{k}}_{\text{B}}\text{T}\;{\text{nm}}^{-1}$) [29,30], *α* ~ 1 . 26nm, is the inverse decay length, *δ* = 1nm, specifies the radius of the repulsive core of DNA, *r* is the distance between the *I*-th DNA binding site and the *k*-th site on the histone. The excluded volume effect between DNA and histone is considered by a half-harmonic potential, i.e., for $r<|a|+r_{0}$,


$$\begin{array}{c} U_{ex}=\frac{1}{2}k{\left(|a|+r_{0}-R\right)}^{2} \end{array}$$ (3)

where $k=8\;{\text{k}}_{\text{B}}\text{T}\;\cdot \;\;{\text{nm}}^{-1}$ is the rigidity coefficient,  | *a* | = 4 . 5nm is the radius of the histone, $r_{0}=1\;\text{nm}$ is the radius of DNA and *R* is the distance between the *I*-th DNA binding site and the center of the histone.

The Brownian dynamics (BD) can be performed using an integration, the second-order BD algorithm [35,37]. A tentative first-order displacement is


$$\begin{array}{cccc} r_{i}^{\prime }(t+\Delta t)-r_{i}(t) & =\sum D_{ij}(t)\frac{F_{j}(t)}{k_{B}T}\Delta t+R_{i}\; & & \;\\ \varphi_{i}^{\prime }(t+\Delta t)-\varphi_{i}(t) & =D_{rot}(t)\frac{T_{i}^{t}(t)}{k_{B}T}\Delta t+\Phi_{i},\; & \text{(4)}\; \end{array}$$

and the final half-step is


$$\begin{array}{cccc} r_{i}(t+\Delta t)-r_{i}^{\prime }(t+\Delta t) & =\sum D_{ij}(t)\frac{-F_{j}(t)+F_{j}^{\prime }(t+\Delta t)}{2k_{B}T}\Delta t\; & & \;\\ \varphi_{i}(t+\Delta t)-\varphi_{i}^{\prime }(t+\Delta t) & =D_{rot}(t)\frac{-T_{q,i}(t)+T_{q,i}^{\prime }(t+\Delta t)}{2k_{B}T}\Delta t,\; & \text{(5)}\; \end{array}$$

where $r_{i}^{\prime }$ is the tentative position, $D_{ij}$ is the Rotne-Prager tensor component [35], and for the computational efficiency, we chose $D_{ij}=D\delta_{ij}=\delta_{ij}k_{B}T∕6\pi \eta R_{H}$, and here $R_{H}$ is effective radius of each segment [37,39], $F_{i}$ and $F_{i}^{\prime }$ are the forces on the *i*-th vertex corresponding to the conformations *r* and $r^{\prime }$, respectively, $T_{q,i}$ and $T_{q,i}^{\prime }$ are the torques on the *i*-th segment; $D_{rot}=k_{B}T∕4\pi \eta R_{d}^{2}l_{0}$ is the rotational diffusion constant, here $R_{d}$ is the diffusion radius of DNA, *R* and *Φ* are the thermal noises with the properties  ⟨ *R* ⟩ = 0,  ⟨ *R* ⊗ *R* ⟩ = 2*DΔt*,  ⟨ *Φ* ⟩ = 0, $\langle \Phi ⊗\Phi \rangle =2D_{rot}\Delta t$.

The center of mass of the histone protein moves following the Langevin equation under the protein-DNA interactions $U_{DH}+U_{ex}$,


$$\begin{array}{c} {\dot{R}}_{COM}=-\frac{1}{\zeta }\nabla_{R_{COM}}(U_{DH}+U_{ex})+\sqrt{\frac{2\;{\text{k}}_{\text{B}}\text{T}}{\zeta }}w \end{array}$$ (6)

where *ζ* = 6*πη* | *a* |  is the the friction coefficient, with *η* the solution viscosity and  | *a* |  the radius of the spherical protein, and *w* ( *t* )  is the Gaussian noise with zero mean and unit variance. It should be noted that the BD (Eq. 6) can be performed using the second-order BD algorithm.

The orientational or rotational degrees of the histone core caused by thermal fluctuations and interactions are modeled by partitioning the rotations into the spatial rotation of the unit axial vector *A* (axial vector of the helical path shown as Fig 1), $\Delta \Omega_{A}$ and the spinning about *A*, *Ψ* [40]. Accordingly, the protein angular Langevin dynamic equations are formulated as (details in S1 Text).


$$\begin{array}{c} A\times {\dot{\Omega }}_{A}|a|=-\frac{1}{\zeta_{rot}}\underset{i}{\sum }(a\times F_{i})\times A∕|a|+\sqrt{\frac{2\;{\text{k}}_{\text{B}}\text{T}}{\zeta_{rot}}}(A\times e_{\Theta }w_{1}+A\times e_{\Phi }w_{2}) \end{array}$$ (7)

$$\begin{array}{c} A\cdot \dot{\Psi }|a|=-\frac{1}{\zeta_{rot}}\underset{i}{\sum }(a\times F_{i})\cdot A∕|a|+\sqrt{\frac{2\;{\text{k}}_{\text{B}}\text{T}}{\zeta_{rot}}}(w_{3}) \end{array}$$ (8)

where $\zeta_{rot}=8\pi \eta |a|$ is the friction due to rotation of the protein in solution, $w_{1}$, $w_{2}$ and $w_{3}$ are the Gaussian noises with zero mean and unit variance. The source code is available at: https://figshare.com/s/b38438c966505126910e.

DNA supercoil can be quantified by linking number *Lk* and its two components, twisting number (*Tw*) and writhing number (*Wr*). A relaxed B-DNA consisting of *N* base pairs has a linking number $Lk_{0}=N∕10.5$. The excess DNA supercoils can be introduced by some enzymes, e,g. gyrase, Topo II, RNA and DNA polymerizes, or by rotating magnetic beads in single-molecule techniques. The resulting linking number difference is then $\Delta Lk\equiv Lk\;-\;Lk_{0}$. The components, excess twisting number (*ΔTw*) and writhing number (*Wr*) follow *ΔLk* = *ΔTw* + *Wr* [41,42] (see examples in S2 Text). For the current model, *ΔTw* can be calculated as the sum of the twisting between neighbor segments of DNA


$$\begin{array}{c} \Delta Tw=\frac{1}{2\pi }\underset{i}{\sum }\theta_{i} \end{array}$$ (9)

*Wr* characterizes the spatial torsion of supercoiled DNA in terms of the sum of the crossings of all DNA segment pairs [43],


$$\begin{array}{c} Wr=\frac{1}{4\pi }\underset{i\neq j}{\sum }\frac{\left(r_{j+1}-r_{j})\times (r_{i+1}-r_{i})\cdot (r_{j}-r_{i}\right)}{|r_{j}-r_{i}{|}^{3}} \end{array}$$ (10)

## Results

### Spontaneously wrapping of DNA around histone

**Fig 2:**
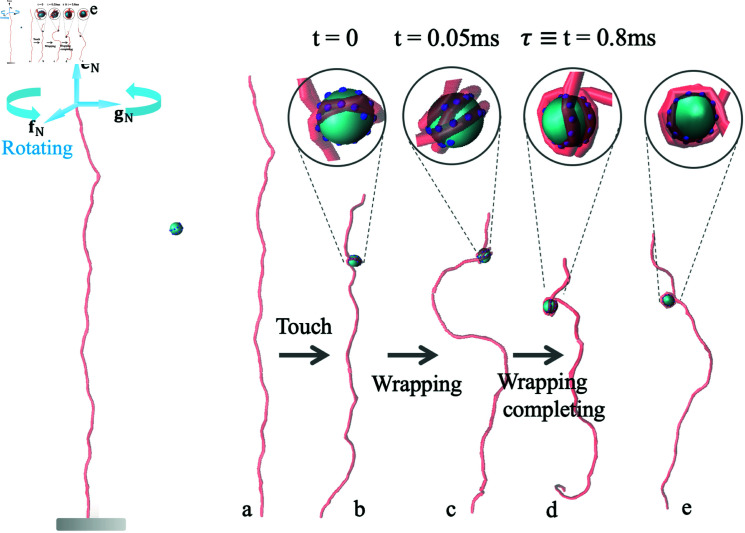
Generation of initial supercoiled DNA and representative snapshots of DNA winding around a histone core. (Left) The initial DNA supercoils were introduced by rotating the base vectors $f_{N}$ and $g_{N}$ about $e_{N}$ attached to the end DNA segment. A single histone core interacts with a positively supercoiled DNA (*ΔLk* = 3) at length of 1000 bp (100 segments) under tension *f* = 0 . 3pN. A complete simulation trajectory includes the sequential steps: histone core searching the DNA chain(a), the histone touching the DNA(b), DNA winding around histone (c) and finally completing the nucleosome wrapping (d and e). The first passage time (*τ*) of nucleosome-wrapping is defined by the time interval between (b) and (d).

Similar to the supercoil turns added by magnets in experiments [14,44], the initial DNA conformation $\left\{r_{i},f_{i},g_{i},e_{i}\right\}$ with a given *ΔLk* was obtained by rotating the end vectors $f_{N},g_{N}$ about $e_{N}$ at rate  � 5 *turns* ∕ *s* while torsionally constraining the other end (Fig 2), where  +  denotes counterclockwise rotation.As the excess linking number reaches to the expected value, the rotation is stalled. In Fig 2, an extended and supercoiled DNA (*ΔLk* = 3) at length 1000*bp* stretched by *f* = 0 . 3pN interacts with a histone. The interactions between DNA and histone with absorption energy $\epsilon =5\;{\text{k}}_{\text{B}}\text{T}$ can wind the DNA around the histone core, forming partially wrapped and mis-wrapped structures, and finally trigger the completion of the nucleosome wrapping. We accordingly define the first passage time (FPT) as the interval from the touching between the DNA chain and the histone to the completion of the nucleosome-wrapping.

The DNA winding in the single nucleosome contributes to approximately  - 1 linking number difference, and consequently induces an extra *ΔΔLk* = + 1 to the unconstrained DNA, which is energetically adverse. However, our simulation suggests that the positive supercoil is able to wrap a histone to form nucleosome.

### Strong interactions between histone and DNA promote the nucleosome wrapping

**Fig 3:**
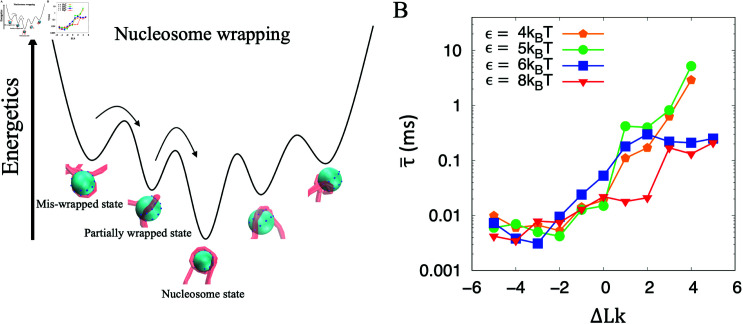
Nucleosome-wrapping driven by interactions between DNA and histone. (A) Schematic illustration of the nucleosome wrapping process. Before completely nucleosome wrapping (Energetic minimum), the system explores a vast number of conformations of the mis-wrapped state (out of left-handed helix path) and partially wrapped state. (B) The mean first passage time (MFPT) for the completion of the nucleosome wrapping depends on the interaction energy strength *ϵ* and the supercoil degree. Each dot was obtained from 20 simulations trajectories with 10*ms* of each (It should be noted that no data was captured for *ΔLk* = 5 and *ϵ* = 4, $5\;{\text{k}}_{\text{B}}\text{T}$ within 10 *ms*). For each given linking number, the DNA is stretched under constant force *f* = 0 . 3pN.

The DNA winding around the histone core is energetically costly. Hence, the interactions between the DNA and histone should be the primary factor for nucleosome wrapping. The conformations exhibited by the histone-DNA complex can be broadly categorized into three types: mis-wrapped (non-left-handed), partially wrapped and nucleosome wrapped states (Fig 3A). The typical pathways of the nucleosome wrapping are *mis-wrapped state* → *partially wrapped state* → *nucleosome state* and *partially wrapped state* → *nucleosome state* (see the S3 Text for the details).

Intuitively, the strong interactions can promote the completion of the nucleosome wrapping. This is confirmed by our simulations of spontaneously nucleosome-wrapping with different DNA supercoils  - 5 ≤ *ΔLk* ≤ 5 and different adsorption energy strengths *ϵ* ($4\;{\text{k}}_{\text{B}}\text{T},5\;{\text{k}}_{\text{B}}\text{T},6\;{\text{k}}_{\text{B}}\text{T}$ and $8\;{\text{k}}_{\text{B}}\text{T}$, corresponding to the adsorption energy density from $6\;{\text{k}}_{\text{B}}\text{T}\;{\text{nm}}^{-1}$ to $11\;{\text{k}}_{\text{B}}\text{T}\;{\text{nm}}^{-1}$) under stretching force *f* = 0 . 3pN (Fig 3B). The interactions from weak to strong correspond to the salt concentrations from high to low. Under all the conditions, both of the positively and negatively supercoiled DNAs can wrap around the histone to form nucleosome. Notably, the strong adsorption energy strength and the presence of negative supercoils serve to augment the efficiency of nucleosome wrapping.

### Nucleosome-wrapping prefers negative supercoils

**Fig 4:**
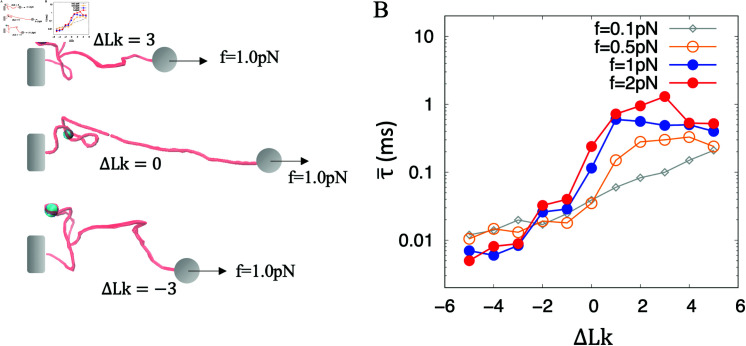
Preference for negative supercoiling. (A) Schematics for a single histone interacting with a supercoiled DNA under stretching force *f* = 1 . 0pN. (B) The MFPT for completing nucleosome wrapping with $\epsilon =6\;{\text{k}}_{\text{B}}\text{T}$ is supercoiling-dependent and force-dependent. Each dot was obtained from 20 simulations trajectories with 10 *ms* of each.

During transcription, the positive supercoils generated by the polymerases could dissociate the nucleosomes, while the negative supercoils behind might facilitate the re-assembly of the nucleosomes [9,10]. The transcription elongation can be stalled by the torque (about 5 ~ 11pN ⋅ nm) induced by the generated twin supercoils if the applied force is larger than 0.5 pN [33,45].If the applied force is larger than the characteristic force ( ~ 0 . 08pN), DNA can be extended [46]. Accordingly, we identify the forces larger than 0.08 pN and smaller than 0.5 pN as the small forces and define the forces larger than 0.5 pN as tensional forces. We have demonstrated that the nucleosome-wrapping process shows a strong preference for negative supercoils under a small force (Fig 3(B)). The efficiency of nucleosome-wrapping is supposed to be force-dependent. Hence, we have compared the mean first passage time (MFPT) of nucleosome wrapping for supercoiled DNAs under different forces. (Fig 4).

For all the cases of the applied forces, the MFPT for nucleosome wrapping by negative supercoiled DNA is significantly shorter compared to that achieved by positively supercoiled DNA. In addition, the applied force can amplify the preference. Indeed, for the case of *f* = 1 . 0pN and 2 . 0pN, the efficiency of nucleosome wrapping under negative supercoils is over two orders of magnitude higher than that under positive supercoils. In comparison, for the small force *f* = 0 . 1pN, the efficiency of nucleosome wrapping under negative supercoils is one order of magnitude higher than that under positive supercoils.

### Nucleosome tends to assemble in proximity to the original nucleosome

**Fig 5:**
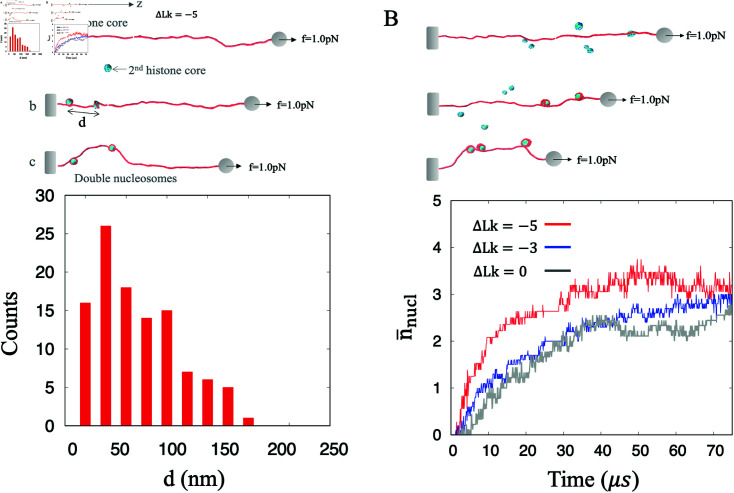
Multiple nucleosomes assembling. (A)Distribution of nucleosome assembling distance along a DNA chain at length of 1000 *bp*. The first nucleosome is constrained at *z* = 20nm, and then the second histone is wound by DNA to form a new nucleosome. The second nucleosome tends to successfully assemble near the first nucleosome. The distribution of the distance *d* between two nucleosomes was obtained from 100 simulation trajectories of 100 *μs* each. (B)Average number of nucleosomes. An initially naked DNA chain with different linking numbers interact with 6 histone cores and form multiple nucleosomes. Compared with the DNA without supercoil (*ΔLk* = 0), the highly supercoiled DNA (*ΔLk* = - 5) enhances efficiency of the nucleosome assembling. Each line is the average over 10 simulation trajectories with 75 *μs* of each.

Our model can be directly extended to the case of multiple nucleosomes on the same DNA template. We investigated how the distance between nucleosomes affects the nucleosome-wrapping. In Fig 5A, the first histone core is wrapped by DNA and pinned at *z* = 20nm. The end of the DNA chain under *ΔLk* = - 5 is stretched by *f* = 1 . 0pN. Then the second histone randomly collides with any site along the DNA chain. Finally, the histone core interacts with DNA and form the second nucleosome. We performed the simulations and calculated the assembling distance *d* between the centers of the two histone cores. We noticed that the second histone tends to complete the assembly near the first one. The assembling probability is 0 if *d* < 10nm due to volume excluding effect between the two histones. The most probable distance for nucleosome assembling is within 30nm < *d* < 50nm, which may results from the substantial DNA bending induced by the first nucleosome.

Besides, we performed simulations of an initially naked DNA chain under *ΔLk* = 0, - 3 or  - 5 simultaneously interacting with 6 histone cores as shown in Fig 5B. Our simulations shows that the histones are simultaneously wrapped by the same DNA and the average number of the nucleosomes increases with time. The average number of the nucleosomes on DNA with *ΔLk* = - 5 reaches to ${\overset{ñ}{n}}_{nucl}=2$ in 10 *μ*s. In comparison, ${\overset{ñ}{n}}_{nucl}=2$ for DNA with *ΔLk* = 0 takes about 30 *μ*s. As above, the nucleosome wrapping favors negative supercoils. Indeed, as one nucleosome wrapping completes, *ΔΔLk* = + 1 is induced on the unconstrained DNA, which in turn reduces the efficiency of following nucleosome assembling.

## Discussion and conclusion

**Fig 6:**
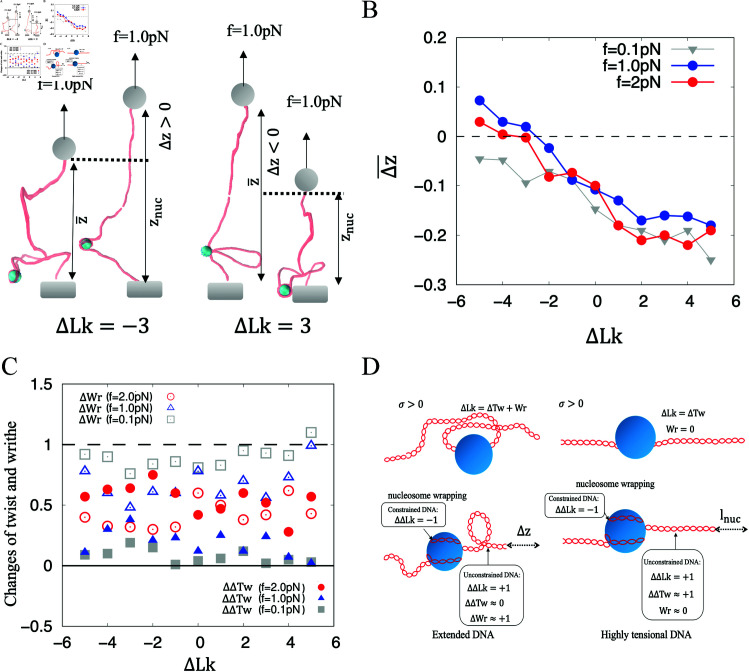
Extension, twist and writhe changes of DNA during nucleosome-wrapping. (A) The extension increment of the supercoiled DNA due to the nucleosome wrapping.Under tensional forces, the nucleosome wrapping increases the extension of the negatively supercoiled DNA while reducing the extension of the positively supercoiled DNA. (B) The mean extension increment resulting from nucleosome wrapping is a function of supercoil and stretching force. The positive increment in DNA extension due to nucleosome wrapping causes the decrease of the potential energy of the extended DNA, which is energetically favored. (C) Changes in the twist (*ΔTw*) and writhe (*Wr*) of the unconstrained DNA due to nucleosome-wrapping. (D)The changes in twist and writhe of (positive supercoils) DNA during winding around histone core. (Left) Extendedly (+) supercoiled DNA wraps around a histone under small force, e.g, *f* = 0 . 1pN. The induced  + 1 linking number is mainly attributed to writhe, which causes work done during nucleosome wrapping. (Right) Highly tensional (+) supercoiled DNA wraps around a histone under large force, e.g, *f* = 10pN. The induced  + 1 linking number is mainly attributed to twist, which increases twisting energy during nucleosome wrapping.

We examined the roles of DNA extension, twist and writhe played in nucleosome wrapping. Our simulations reveal that the change in DNA extension due to nucleosome-wrapping, $\Delta z\equiv (z_{nuc}-\tilde{z})/L$, where *L* = 340 nm, depends on the supercoiling (see Fig 6A). The supercoiling-dependent $\Delta z^{\left(+\right)}<\Delta z^{\left(-\right)}$ (Fig 6B), leads to the work done being $-f\Delta z^{\left(+\right)}>-f\Delta z^{\left(-\right)}$. The smaller work done by force *f* results in a reduced energy barrier for nucleosome wrapping. Therefore, nucleosome wrapping tends to occur preferentially for negatively supercoiled DNA.

For extendedly supercoiled DNA under *f* = 0 . 1pN, the induced *ΔΔLk* = + 1 on the unconstrained DNA is primarily attributed to writhe, with *ΔWr* ≈ + 1 and *ΔΔTw* ≈ 0 (Fig 6C), where $\Delta Wr\;\equiv \;Wr^{un}(\tau )\;-\;Wr^{un}(0)$, $\Delta \Delta Tw\;\equiv \;\Delta Tw^{un}(\tau )\;-\;\Delta Tw^{un}(0)$, *ΔΔLk* = *ΔΔTw* + *ΔWr* based on Eqs 9 and 10 and the superscript *un* stands for the unconstrained DNA. The increase in writhe rather than the change in twisting energy governs the dynamics. In other words, for positive writhe,  | *Wr* |  increases due to nucleosome-wrapping. In contrast, for negative writhe,  | *Wr* |  decreases due to nucleosome wrapping. The negative supercoiling results in comparatively lower amount of work-done by *f*, implying a decreased energy barrier for nucleosome wrapping.

In Fig 6(C), we observed that as the force *f* increases to 2.0 pN, there is a corresponding increase in *ΔΔTw* and a decease in *ΔWr*. For highly tensional DNA, such as those subjected to *f* = 5 , 10 and 20pN, which are commonly utilized for stretching chromatin [12,14], however, the increment of twist *ΔΔTw* ≈ 1 meanwhile writhe *ΔWr* ≈ 0 during nucleosome forming, giving a rise to a supercoiling-independent change in extension, $\Delta z=l_{nuc}$. The change of the twisting energy is supercoiling-dependent, i.e., $\Delta E_{\pm }=\frac{1}{2}C[{\left(\Delta Lk\;\pm \;1\right)}^{2}\;-\;{\left(\Delta Lk\right)}^{2}]$, where $C\equiv k_{B}Tl_{t}(L\;-\;l_{nuc})\Omega_{0}^{2}∕{\left(Lk_{0}\right)}^{2}$ is the stiffness of the whole unconstrained DNA [38]. Evidently, $\Delta E_{+}>0>\Delta E_{-}$, meaning nucleosome-wrapping always prefers negative supercoils.

In both of the cases, the energy barrier associated with nucleosome wrapping under positively supercoiled DNA exceeds that of negatively supercoiled DNA, thereby the preference for negative supercoiling (Fig 6D).

In eukaryotic chromatin, DNA attains a highly compact structure due to its wrapping around histone cores, contributing to negative supercoiling. To elucidate the mechanism underlying the spontaneous wrapping of supercoiled DNA around histone proteins, we designed a Brownian dynamics model of the DNA-histone complex. We demonstrated that both the positively and negatively supercoiled DNAs can form the nucleosome conformation with appropriate absorption energies between DNA and histone. Furthermore, we observed that the preference for negative supercoiling during nucleosome wrapping becomes more evident with increasing force. Finally, our model has been extended to the case of multiple nucleosomes on the same DNA template and found that nucleosome favors assembling near the original nucleosome. Several improvements to our model are warranted for future studies. The first one is the over-twist on the constrained DNA that wraps around histone, which may mainly originates from interaction with the histone rather than DNA intrinsic property [47]. Secondly, the nucleosome filament requires histone H1, which associates with the entry and exit of DNA from the histone core [21,24]. The incorporation of the effects of the histone H1 allows nucleosomes to be arranged into a compact and stabilized array. In the large force regime, the dissociation of histone from DNA is dominating over re-assembling of nucleosome. Consequently, the net nucleosomes reassembling rate is determined by the difference between re-assembling rate and dissociate rate. Thus, the force-dependent kinetics of the nucleosome dissociation and re-assembling presents a promising research topic in the future. Finally, using some manipulations to supercoiled DNA and protein-DNA complexes, we can improve our model to study more intriguingly experimental and biological processes, e.g, DNA manipulations by magnetic beads in experiments and by certain enzymes that introduce supercoils to DNA, such as RNA polymerases, gyrase, and Topo II.

## Supporting information

S1 Text Dynamics of the rotational degrees. (PDF)

S2 Text Pathway of nucleosome wrapping and the intermediate states. (PDF)

S3 Text Quantitative measures of DNA supercoiling. (PDF)

## References

[1] Bates AD, Maxwell A, Maxwell T. DNA topology. Oxford: Oxford University Press; 2005.

[2] Kornberg RD. Structure of chromatin. Annu Rev Biochem. 2003:46(1);931. 10.1146/annurev.bi.46.070177.004435 332067

[3] Arents G, Burlingame R, Wang B, Love W, Moudrianakis E. The nucleosomal core histone octamer at 3.1 A resolution: a tripartite protein assembly and a left-handed superhelix. Proc Natl Acad Sci U S A. 1991;88(22):10148–52. 1946434 10.1073/pnas.88.22.10148 PMC52885

[4] Luger K, Mäder AW, Richmond RK, Sargent DF, Richmond TJ. Crystal structure of the nucleosome core particle at 2.8 A resolution. Nature. 1997;389(6648):251–60. doi: 10.1038/38444 9305837

[5] Song F, Chen P, Sun D, Wang M, Dong L, Liang D, et al. Cryo-EM study of the chromatin fiber reveals a double helix twisted by tetranucleosomal units. Science 2014;344(6182):376–80. doi: 10.1126/science.1251413 24763583

[6] Hamkalo BA. Chromatin: structure and function. NATO Adv Study Inst. 2006;9(4):7–14.

[7] Kornberg R, Lorch Y. Twenty-five years of the nucleosome, fundamental particle of the eukaryote chromosome. 1999. 10.1016/s0092-8674(00)81958-3 10458604

[8] Corless S, Gilbert N. Effects of DNA supercoiling on chromatin architecture. Biophys Rev 2016;8(3):245–58. doi: 10.1007/s12551-016-0210-1 27738453 PMC5039215

[9] Kouzine F, Sanford S, Elisha-Feil Z, Levens D. The functional response of upstream DNA to dynamic supercoiling in vivo. Nat Struct Mol Biol 2008;15(2):146–54. doi: 10.1038/nsmb.1372 18193062

[10] Lavelle C. Pack, unpack, bend, twist, pull, push: the physical side of gene expression. Curr Opin Genet Dev. 2014;2574–84. doi: 10.1016/j.gde.2014.01.001. 24576847

[11] Allemand J-F, Bensimon D, Croquette V. The elasticity of a single supercoiled DNA molecule (vol 271, pg 1835, 1996). Science. 1996:272(5263);797. 10.1126/science.271.5257.1835 8596951

[12] Cui Y, Bustamante C. Pulling a single chromatin fiber reveals the forces that maintain its higher-order structure. Proc Natl Acad Sci U S A 2000;97(1):127–32. doi: 10.1073/pnas.97.1.127 10618382 PMC26627

[13] Le TT, Gao X, Park SH, Lee J, Inman JT, Lee JH, et al. Synergistic coordination of chromatin torsional mechanics and topoisomerase activity. Cell. 2019;179(3):619-631.e15. doi: 10.1016/j.cell.2019.09.034 31626768 PMC6899335

[14] Lee J, Wu M, Inman JT, Singh G, Park SH, Lee JH, et al. Chromatinization modulates topoisomerase II processivity. Nat Commun 2023;14(1):6844. doi: 10.1038/s41467-023-42600-z 37891161 PMC10611788

[15] Sakaue T, Yoshikawa K, Yoshimura SH, Takeyasu K. Histone core slips along DNA and prefers positioning at the chain end. Phys Rev Lett 2001;87(7):078105. doi: 10.1103/PhysRevLett.87.078105 11497924

[16] Mohammad-Rafiee F, Kulić IM, Schiessel H. Theory of nucleosome corkscrew sliding in the presence of synthetic DNA ligands. J Mol Biol 2004;344(1):47–58. doi: 10.1016/j.jmb.2004.09.027 15504401

[17] Mochrie S, Mack A, Schlingman D, Collins R, Kamenetska M, Regan L. Unwinding and rewinding the nucleosome inner turn: force dependence of the kinetic rate constants. Phys Rev E. 2013:87(1);012710. 10.1103/PhysRevE.87.012710 PMC3902847 23410362

[18] Stevens MJ, Kremer K. The nature of flexible linear polyelectrolytes in salt free solution: a molecular dynamics study. J Chem Phys 1995;103(4):1669–90. doi: 10.1063/1.470698 1.470698

[19] Fujiwara S, Sato T. Molecular dynamics simulations of structural formation of a single polymer chain: bond-orientational order and conformational defects. J Chem Phys 1997;107(2):613–22. doi: 10.1063/1.474421 1.474421

[20] Noguchi H, Yoshikawa K. Morphological variation in a collapsed single homopolymer chain. J Chem Phys 1998;109(12):5070–7. doi: 10.1063/1.477121

[21] Biswas M, Voltz K, Smith JC, Langowski J. Role of histone tails in structural stability of the nucleosome. PLoS Comput Biol 2011;7(12):e1002279. doi: 10.1371/journal.pcbi.1002279 22207822 PMC3240580

[22] Kenzaki H, Takada S. Partial unwrapping and histone tail dynamics in nucleosome revealed by coarse-grained molecular simulations. PLoS Comput Biol 2015;11(8):e1004443. doi: 10.1371/journal.pcbi.1004443 26262925 PMC4532510

[23] Lequieu J, Córdoba A, Schwartz DC, de Pablo JJ. Tension-dependent free energies of nucleosome unwrapping. ACS Cent Sci 2016;2(9):660–6. doi: 10.1021/acscentsci.6b00201 27725965 PMC5043429

[24] Luque A, Ozer G, Schlick T. Correlation among DNA linker length, linker histone concentration, and histone tails in chromatin. Biophys J 2016;110(11):2309–19. doi: 10.1016/j.bpj.2016.04.024 27276249 PMC4906253

[25] Parsons T, Zhang B. Critical role of histone tail entropy in nucleosome unwinding. J Chem Phys 2019;150(18):185103. doi: 10.1063/1.5085663 31091895

[26] Noguchi H, Yoshikawa K. Folding path in a semiflexible homopolymer chain: a Brownian dynamics simulation. J Chem Phys 2000;113(2):854–62. doi: 10.1063/1.481861 1.481861

[27] Li W, Dou S-X, Wang P-Y. Brownian dynamics simulation of nucleosome formation and disruption under stretching. J Theor Biol 2004;230(3):375–83. doi: 10.1016/j.jtbi.2004.03.028 15321707

[28] Li W, Dou S-X, Wang P-Y. The histone octamer influences the wrapping direction of DNA on it: Brownian dynamics simulation of the nucleosome chirality. J Theor Biol 2005;235(3):365–72. doi: 10.1016/j.jtbi.2005.01.016 15882698

[29] Li W, Dou S-X, Xie P, Wang P-Y. Brownian dynamics simulation of directional sliding of histone octamers caused by DNA bending. Phys Rev E Stat Nonlin Soft Matter Phys. 2006;73(5 Pt 1):051909. doi: 10.1103/PhysRevE.73.051909 16802969

[30] Wocjan T, Klenin K, Langowski J. Brownian dynamics simulation of DNA unrolling from the nucleosome. J Phys Chem B. 2009;113(9):2639–46 19708203 10.1021/jp806137e

[31] Higuchi Y, Sakaue T, Yoshikawa K. How does torsional rigidity affect the wrapping transition of a semiflexible chain around a spherical core? Physics. 2010;82(3):336–54. 10.1103/PhysRevE.82.031909 21230110

[32] Dobrovolskaia IV, Arya G. Dynamics of forced nucleosome unraveling and role of nonuniform histone-DNA interactions. Biophys J 2012;103(5):989–98. doi: 10.1016/j.bpj.2012.07.043 23009848 PMC3433614

[33] Ma J, Bai L, Wang MD. Transcription under torsion. Science 2013;340(6140):1580–3. doi: 10.1126/science.1235441 23812716 PMC5657242

[34] Seol Y, Neuman KC. The dynamic interplay between DNA topoisomerases and DNA topology. Biophys Rev. 2016;8(Suppl 1):101–11. doi: 10.1007/s12551-016-0240-8 28510219 PMC5418509

[35] Klenin K, Merlitz H, Langowski J. A Brownian dynamics program for the simulation of linear and circular DNA and other wormlike chain polyelectrolytes. Biophys J. 1998;74(2 Pt 1):780–8. doi: 10.1016/S0006-3495(98)74003-2 9533691 PMC1302559

[36] Vologodskii A. Brownian dynamics simulation of Knot diffusion along a stretched DNA molecule. Biophys J. 2006;90(5):1594–7. 16361333 10.1529/biophysj.105.074682 PMC1367310

[37] Ivenso ID, Lillian TD. Simulation of DNA supercoil relaxation. Biophys J 2016;110(10):2176–84. doi: 10.1016/j.bpj.2016.03.041 27224483 PMC4880802

[38] Wan B, Yu J. Two-phase dynamics of DNA supercoiling based on DNA polymer physics. Biophys J 2022;121(4):658–69. doi: 10.1016/j.bpj.2022.01.001 35016860 PMC8873955

[39] Mielke SP, Fink WH, Krishnan VV, Grønbech-Jensen N, Benham CJ. Transcription-driven twin supercoiling of a DNA loop: a Brownian dynamics study. J Chem Phys 2004;121(16):8104–12. doi: 10.1063/1.1799613 15485274

[40] Al Masri C, Wan B, Yu J. Nonspecific vs. specific DNA binding free energetics of a transcription factor domain protein. Biophys J 2023;122(22):4476–87. doi: 10.1016/j.bpj.2023.10.025 37897044 PMC10722393

[41] Fuller FB. The writhing number of a space curve. Proc Natl Acad Sci U S A 1971;68(4):815–9. doi: 10.1073/pnas.68.4.815 5279522 PMC389050

[42] Fuller FB. Decomposition of the linking number of a closed ribbon: a problem from molecular biology. Proc Natl Acad Sci U S A 1978;75(8):3557–61. doi: 10.1073/pnas.75.8.3557 16592550 PMC392823

[43] Klenin K, Langowski J. Computation of writhe in modeling of supercoiled DNA. Biopolymers 2000;54(5):307–17. doi: 10.1002/1097-0282(20001015)54:5<307::AID-BIP20>3.0.CO;2-Y 10935971

[44] van Loenhout MTJ, de Grunt MV, Dekker C. Dynamics of DNA supercoils. Science 2012;338(6103):94–7. doi: 10.1126/science.1225810 22983709

[45] Ma J, Tan C, Gao X, Fulbright RM, Roberts JW, Wang MD. Transcription factor regulation of RNA polymerase’s torque generation capacity. Proc Natl Acad Sci U S A 2019;116(7):2583–8. doi: 10.1073/pnas.1807031116 30635423 PMC6377492

[46] Marko JF, Siggia ED. Stretching DNA. Macromolecules. 1995;28(26):8759–70.

[47] Hayes JJ, Bashkin J, Tullius TD, Wolffe AP. The histone core exerts a dominant constraint on the structure of DNA in a nucleosome. Biochemistry 1991;30(34):8434–40. doi: 10.1021/bi00098a022 1653013

