# Supplementary material for: DNA spontaneously wrapping around a histone core prefers negative supercoiling: A Brownian dynamics study
Source: PLoS Comput Biol. 2025 Jan 28;21(1):e1012362. doi: 10.1371/journal.pcbi.1012362 (PMC11793753; doi:10.1371/journal.pcbi.1012362)
Supplement: S1 Text — (PDF) [file pcbi.1012362.s001.pdf]

S1.DYNAMICS OF THE ROTATIONAL DEGREES

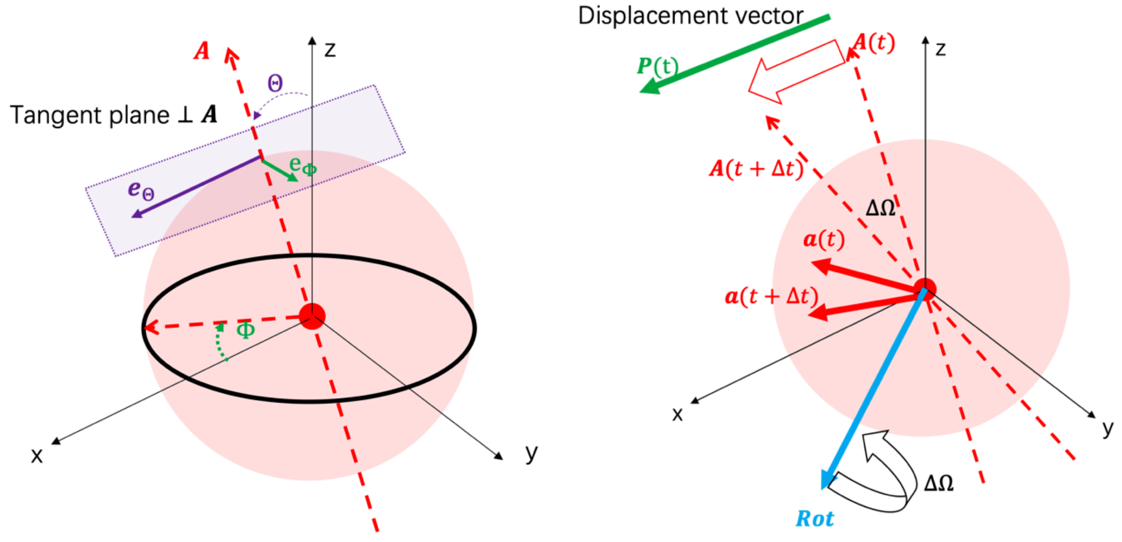

(a) (b)

FIG. 1: (A)Spatial rotation of the axial vector of the spherical protein, **A**, described by polar angle Θ (relative to the z-axis) and azimuthal angle Φ (relative to the x-axis). The tangent plane of the sphere perpendicular to **A** is uniquely determined, which can be spanned by $\mathbf{e}_\Theta$ and $\mathbf{e}_\Phi$ (B) Rotation of the axis **A** without spinning. The projection of the forces and the fluctuations together onto the tangent plane cause the rotation of the axis **A**. The displacement unit-vector of **A**, **P** and **A** define the rotational axis **Rot**. An arbitrary vector **a** rotates about **Rot** by angle ΔΩ.

The resultant force exerted at the center of mass causes the translational displacement. The 3-dimensional rotation can be decomposed into 2 independent operations, axial rotation (FIG.7) and spinning (FIG 8) about its axis. The above dynamics can be briefly summarized as the compact formula EQ.7&8. EQ. 7 describes the rotation of the axial vector **A**. The rotation of **A** is spanned by polar angles ΔΘ and ΔΦ, i.e., $\Delta\Omega = \sqrt{\Delta\Theta^2 + \Delta\Phi^2}$. Together, an arbitrary vector **a** rotates about **Rot** by angle ΔΩ. After the rotation operation, the spinning of the sphere carries the vector **a** to rotate about **A** by angle Ψ. The spinning angle Ψ follows the EQ.8.

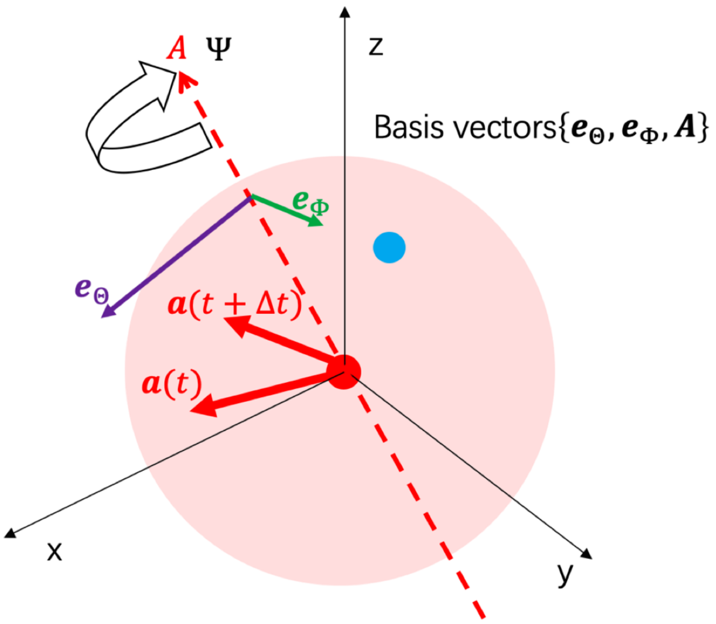

FIG. 2: Spinning about the axis $\mathbf{A}$. The resulting spinning operation is conducted to the basis vectors $\{\mathbf{e}_\Theta, \mathbf{e}_\Phi, \mathbf{A}\}$. Vector $\mathbf{a}$ rotates about $\mathbf{A}$ by angle Ψ.
