# Supplementary material for: DNA spontaneously wrapping around a histone core prefers negative supercoiling: A Brownian dynamics study
Source: PLoS Comput Biol. 2025 Jan 28;21(1):e1012362. doi: 10.1371/journal.pcbi.1012362 (PMC11793753; doi:10.1371/journal.pcbi.1012362)
Supplement: S2 Text — (PDF) [file pcbi.1012362.s002.pdf]

S3. QUANTITIVE MEASURES OF DNA SUPERCOILING

DNA supercoil can be quantified by linking number (Lk), which is further decomposed into twisting number (Tw) and writhing number (Wr). Lk is topological and represents the number of two strands of DNA entwining around each other[1]. Tw counts the number of one of the stands winding around the axial curve of DNA, which is additive. Wr characterizes the spatial torsion of the supercoiled DNA in terms of the average of the crossings of the DNA axial curve. They are mathematically defined as integrations[2, 3]. Fig 1(a) shows examples of untwisted and twisted rods to illustrate the twisting number and a bent and closed circular rod to characterize the writhing number. These quantities follows a constraint $Lk = Tw + Wr$. A relaxed B-DNA consisting of N base pairs has a linking number $Lk_0 = N/10.5$ and a twisting number $Tw_0 = N/10.5$. The excess linking is then defined as $\Delta Lk \equiv Lk - Lk_0$. And the excess twist is $\Delta Tw \equiv Tw - Tw_0$. Thus, the relation $\Delta Lk = \Delta Tw + Wr$ holds for the supercoiled DNA. It should be noted that the non-additivity of ΔLk is attribute to that of Wr. ΔTw and Wr are mathematically defined as $\frac{1}{4\pi}\int ds\theta(s)$ and $\frac{1}{4\pi}\int \frac{d\mathbf{r}_2 \times d\mathbf{r}_1 (\mathbf{r}_2 - \mathbf{r}_1)}{|\mathbf{r}_2 - \mathbf{r}_1|^3}$[1, 3], respectively (Fig 1(b)).

[1] F. B. Fuller, The writhing number of a space curve, Proceedings of the National Academy of Sciences **68**, 815 (1971).

[2] A. D. Bates, A. Maxwell, T. Maxwell, *et al.*, *DNA topology* (Oxford University Press, USA, 2005).

[3] K. Klenin and J. Langowski, Computation of writhe in modeling of supercoiled dna, Biopolymers: Original Research on Biomolecules **54**, 307 (2000).

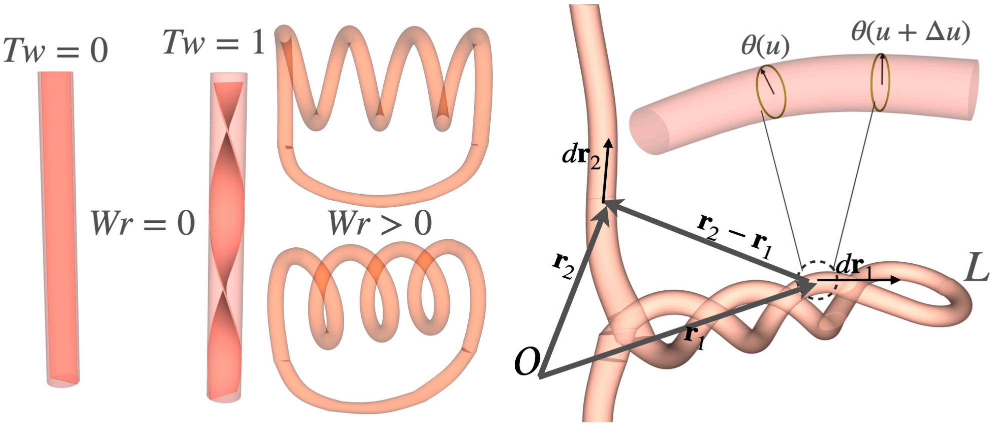

(a) (b)

FIG. 1: Twisting, writhing and linking number. (a)An untwisted and unbent rod, $Tw = 0$,$Wr = 0$ and a rod twisted without bent, $Tw = 1$, $Wr = 0$ and $Lk = 1$. (right) The crossings from two views of the same closed circular rod: $wr = 0$,$wr = 3$. Writhing number is the average of the crossings, wr, from all views, i.e., $Wr \equiv \bar{wr}$. (b) The local twist and the definition of Wr. The vectors $\mathbf{r}_1$ and $\mathbf{r}_2$ represent two different sites along DNA. Wr is defined as $\frac{1}{4\pi}\int \frac{dr_2 \times dr_1 (r_2 - r_1)}{|r_2 - r_1|^3}$, which in general is non-additive, while the local twist $\theta(u + \Delta u) - \theta(u)$ is additive.
