# Supplementary material for: DNA spontaneously wrapping around a histone core prefers negative supercoiling: A Brownian dynamics study
Source: PLoS Comput Biol. 2025 Jan 28;21(1):e1012362. doi: 10.1371/journal.pcbi.1012362 (PMC11793753; doi:10.1371/journal.pcbi.1012362)
Supplement: S3 Text — (PDF) [file pcbi.1012362.s003.pdf]

S3. PATHWAY OF NUCLEOSOME WRAPPING AND THE INTERMEDIATE STATES

Here we introduce an order X to quantitatively characterize the level of left-handed winding of DNA,

$$X \equiv \sqrt{\sum_j (\Gamma(j+1) - \Gamma(j))^2} \tag{1}$$

where the index j denotes the j-th DNA segments which binding to the histone, $\Gamma(j)$ denotes the index of the binding site on the histone to which the j-th segment binds. Hence, for a fully $145bp$ wrapping in nucleosome state, $X \geqslant \sqrt{15}$. Partially wrapped conformations are binding length $\leqslant 140bp$ and $X < \sqrt{15}$. The remaining conformations are all mis-wrapped state (FIG.8).

To reach the fully wrapped state of nucleosome, a vast number of conformations of the DNA and histone complex are explored. A mis-wrapped can re-arrange its segments to approach to a partial and left-handed wrapping, and then completes the fully wrapping. In this process, the positively supercoiled DNA offers right-handed superhelix, which are energetically adverse in nucleosome wrapping. Thus, positive supercoils can provides more mis-wrapped conformations (non-left handed helical wrapping). However, the negatively supercoiled DNA offers left-handed superhelix, which are energetically favored. Consequently, negative supercoils can provides more partially wrapped conformations.

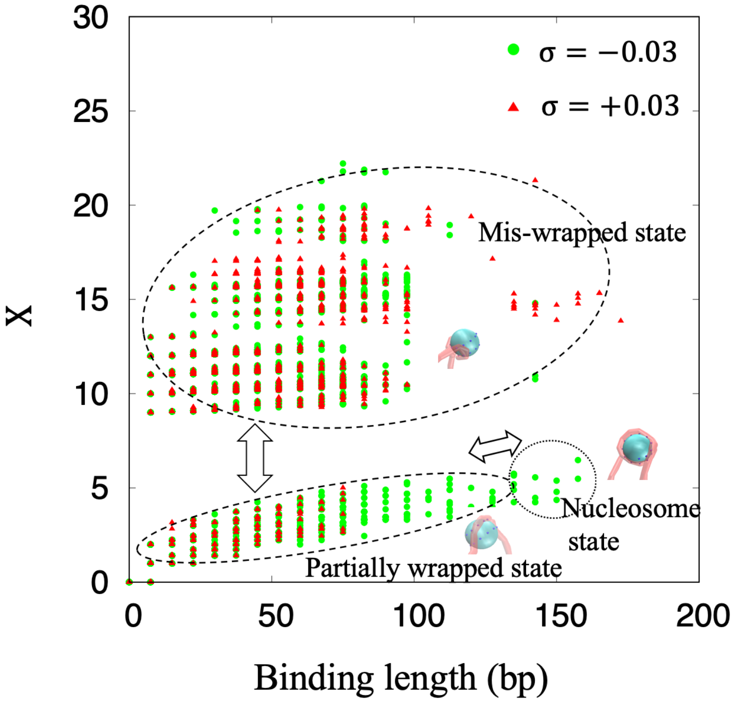

FIG. 1: Negative supercoils offers much more partially wrapped conformations close to the nucleosome state. The wrapping paths can be: *mis-wrapped state*$\rightarrow$*partially wrapped state*$\rightarrow$*nucleosome state* or *partially wrapped staterightarrownucleosome state*. Negative supercoil energetically favors the right-handed helical DNA, which prefers partially wrapped state to mis-wrapped state. In contrast, positive supercoil favors the left-handed helical DNA, which prefers mis-wrapped state to partially wrapped state.
